# Creating a communication system from scratch: gesture beats vocalization hands down

**DOI:** 10.3389/fpsyg.2014.00354

**Published:** 2014-04-29

**Authors:** Nicolas Fay, Casey J. Lister, T. Mark Ellison, Susan Goldin-Meadow

**Affiliations:** ^1^School of Psychology, University of Western AustraliaCrawley, WA, Australia; ^2^Department of Psychology, University of ChicagoChicago, IL, USA

**Keywords:** alignment, gesture, vocalization, multimodal, signs, language origin, embodiment

## Abstract

How does modality affect people's ability to create a communication system from scratch? The present study experimentally tests this question by having pairs of participants communicate a range of pre-specified items (emotions, actions, objects) over a series of trials to a partner using either non-linguistic vocalization, gesture or a combination of the two. Gesture-alone outperformed vocalization-alone, both in terms of successful communication and in terms of the creation of an inventory of sign-meaning mappings shared within a dyad (i.e., sign alignment). Combining vocalization with gesture did not improve performance beyond gesture-alone. In fact, for action items, gesture-alone was a more successful means of communication than the combined modalities. When people do not share a system for communication they can quickly create one, and gesture is the best means of doing so.

## Introduction

And the Lord came down to see the city and the tower which the children of men builded. And the Lord said, “Behold, the people is one, and they have all one language; and this they begin to do: and now nothing will be restrained from them, which they have imagined to do. Go to, let us go down, and there confound their language, that they may not understand one another's speech.” (Genesis 11:5–8, King James Version).

The Book of Genesis tells of the people of Babel, who build a tower that reaches to heaven. God, angered by their arrogance, and concerned by what the people might be capable of, imposes different unshared languages on them, reasoning that without a shared language the people would not be able to communicate, and thus not be able to successfully cooperate. This story was once used to explain the great variety of human languages (approximately 7000 different languages; Lewis, 2009).

Would confounding the language of the people of Babel have stopped them from successfully communicating with one another? This is unlikely. People have successfully established shared communication systems in the absence of a common language. This is seen in pidgins: simple languages that develop among groups who do not share a common language (Thomason and Kaufman, 1988) and in the sign languages that arise when deaf people are brought together (Kegl et al., 1999; Senghas et al., 2004). The present study seeks to determine which communication modality is best suited to establishing a shared communication system from scratch when people are prohibited from using their common language. The question of which modality is best suited to the creation of an *ad hoc* communication system can help inform one of the oldest and most controversial questions in science; the origin of language (Fitch, 2010). In the absence of direct evidence, this question cannot be answered with any certainty. But simulating a scenario in which modern humans must create a new communication system from scratch can help us generate an informed guess. In this paper we use an experimental approach to examine which modality—non-linguistic vocalization, gesture or a combination of non-linguistic vocalization and gesture—best facilitates participants' ability to create a shared communication system with a partner. Specifically, we compare pairs of participants' communication accuracy and the extent to which they use the same signs to communicate the same meanings.

First we review the different theories of the origin of language and evidence supporting each position. Next we review experimental studies of natural spoken language and how they can be extended to deal with novel situations. We then discuss experimental-semiotic studies that examine the genesis of new communication systems when people are prohibited from using their existing language system. Finally, we state the experimental hypotheses and report the results of the present study.

## Vocal, gestural, and multimodal accounts of the origin of language

There are several theories of the origin of language, the most intuitively appealing being that human language developed from non-linguistic vocalizations (MacNeilage, 1998; Cheney and Seyfarth, 2005; Mithen, 2005). Vocalization is our primary means of communication, so it's easy to imagine human language evolving from the vocalizations of non-human primates. Like human speech, the vocalizations of non-human primates can be referential; vervet monkeys produce at least three predator-specific alarm calls that are understood by conspecifics (Seyfarth et al., 1980). However, anatomical and physiological constraints limit the vocal repertoire of non-human primates primarily to a small set of innately specified emotional signals. There is also evidence that non-human primates combine single calls into structurally more complex units with a different meaning, thereby expanding their vocal repertoire (Zuberbühler, 2002; Arnold and Zuberbühler, 2006). For example, when preceded by a low pitched “boom,” the predator alarm calls of Campbell's monkeys are understood by another species, Diana monkeys, to indicate a lower level of direct threat than when the alarm calls are not preceded by a boom (Zuberbühler, 2002). Combinatorial patterning of this kind may have acted as a precursor to syntax. Cheney and Seyfarth (2005) propose that these rudimentary representational abilities are exactly what we'd expect to find in a pre-linguistic ancestor.

This view is challenged by a competing explanation; that language originated through gesture (Hewes, 1973; Corballis, 2003; Arbib, 2005). The brief timeframe in which some new sign languages have become established supports a gesture-first account (Kegl et al., 1999; Sandler et al., 2005). Several other phenomena point to the naturalness of gesture: people of all cultures gesture while they speak (Feyereisen and de Lannoy, 1991); blind people gesture (Iverson and Goldin-Meadow, 1998); speaking adults can successfully adopt gesture as their sole means of communication at the request of experimenters (Goldin-Meadow et al., 1996) or when the environment dictates (e.g., when working in a noisy sawmill; Meissner and Philpott, 1975); many of the lexical items that hearing children produce in the earliest stages of language learning appear first in gesture and only later move to the verbal lexicon (Iverson and Goldin-Meadow, 2005); young deaf children whose profound hearing losses prevent them from acquiring spoken language, and whose hearing parents have not exposed them to sign language, turn to gesture to communicate, and fashion a system of signs, called *homesign*, that contains the fundamental properties of human language (Goldin-Meadow, 2003). Perhaps the most compelling evidence in favor of a gesture-first account is that attempts to teach non-human primates to talk have failed (Hayes, 1952), whereas attempts to teach them a gestural language have been moderately successful (Gardner and Gardner, 1969; Savage-Rumbaugh et al., 1986). This, in addition to the greater flexibility of ape gestures (compared to vocal signals; Pollick and de Waal, 2007), suggests our closest relative is better equipped to communicate by gesture than by speech.

A multimodal account assumes that the earliest forms of language were not restricted to a single modality. Instead, communication occurred by any means available. Bickerton dubs this the “catch-as-catch-can” evolution of language (Bickerton, 2007, p. 512), in which language evolved from whatever rudimentary gestures or sounds were able to communicate meaning effectively. In support of this position it has been observed that, during conversation, bilinguals in a spoken and a signed language often blend their communication across the different modalities (Emmorey et al., 2008), and hearing children produce their first two-element “sentences” in gesture + speech combinations (point at bird + “nap”) and only later produce them entirely in speech (“bird nap”) (Iverson and Goldin-Meadow, 2005; Özçalıþkan and Goldin-Meadow, 2005). Thus, given the opportunity, people use both modalities. Perniss et al. (2010) argue for a multimodal account, pointing out that vocalization-only and gesture-only explanations for language origin are both burdened with explaining why the other form of communication also exists and how it arose. They argue that the neural systems controlling vocalization and gesture are so tightly integrated because these systems have been connected from the beginning (see also Goldin-Meadow and McNeill, 1999).

## Experimental studies: extending spoken language

Acts of reference, in which individuals refer to an object, emotion, action or some other specifiable thing, are ubiquitous to everyday communication. Several tasks have been developed to experimentally examine the referential use of language. In these tasks the experimenter assigns the participants' communicative intentions, whether this involves describing an object or giving directions to a location (for a review see Krauss and Fussell, 1996).

By having participants describe objects that lack a pre-existing name, researchers have examined the process through which people establish joint reference. One participant, the director, communicates a series of abstract shapes from an array to a partner, the matcher, who tries to identify each shape from their array. Interacting partners extend their linguistic system by creating new labels for these novel shapes (e.g., Krauss and Weinheimer, 1964; Clark and Wilkes-Gibbs, 1986). Furthermore, participants' shape descriptions, which initially are elaborate, become increasingly succinct and abstract, such that a shape first described as “Looks like a Martini glass with legs on each side” is referred to as “Martini” over the course of successive references (Krauss and Fussell, 1996, p. 679). Thus, once a shared label has been mutually agreed upon, or grounded, directors use more efficient descriptions that are understood by the matcher. Similar refinement is seen in speech-accompanying gestures (Hoetjes et al., 2011). Interaction is crucial to this process; without it, the referring expressions are longer and more complex (Krauss and Weinheimer, 1966; Hupet and Chantraine, 1992).

Other referential communication tasks show that participants' referring expressions become shared, or aligned, through interaction. Garrod and Anderson (1987) examined the linguistic descriptions used by pairs of participants working together to navigate through a computerized maze. Unlike the shape description task where participant role is typically fixed as either director or matcher, in the maze game both participants give and receive location descriptions (i.e., there is role-switching). Garrod and Anderson (1987) observed that, as the task progressed, pairs of interacting participants increasingly used the same description schemes to communicate locations on the maze. For example, if one participant used a coordinate scheme to communicate a maze location (e.g., “I'm in position A4”) their partner was disproportionately likely to use the same spatial description scheme. Similar interactive alignment is observed for other aspects of linguistic form, including syntax (Branigan et al., 2000) and prosody (Giles et al., 1992). This incremental coupling between production and comprehension processes can explain why conversation is easy: linguistic representations activated by the speaker prime similar representations in the listener, and these representations retain enough activation such that when it is the listener's turn to speak they are reused (and readily understood by the previous speaker; Garrod and Pickering, 2004).

Together, these studies show that language can be rapidly extended to deal with novel situations. They demonstrate that interaction is critical for efficient communication, and that when people alternate speaker and listener roles, they increasingly share, or align upon, the same communication system. Experimental-semiotic studies adopt similar experimental paradigms to study the process through which new communication systems arise and evolve when participants are denied use of their existing linguistic system.

## Experimental studies: creating new communication systems

Because language does not leave fossils, it is difficult to test theories of the origin of language. Moreover, because observational studies of the emergence of pidgins and new sign languages lack experimental control, it is difficult to confidently isolate the variables critical to the genesis and evolution of new languages. Experimental-semiotic studies try to overcome these problems by studying the emergence of new communication systems under controlled laboratory conditions. They do this by creating a situation where participants must communicate without using their existing language system (for a review see Galantucci and Garrod, 2011). Typically, participants communicate in a novel modality, for example, through drawing (Galantucci, 2005; Garrod et al., 2007), through gesture (Goldin-Meadow et al., 1996; Gershkoff-Stowe and Goldin-Medow, 2002; Goldin-Meadow et al., 2008; Langus and Nespor, 2010; Fay et al., 2013) or movement (Scott-Phillips et al., 2009; Stolk et al., 2013), and the experimenters study how communication systems evolve across repeated interactions between the human agents.

**Figure 1:**
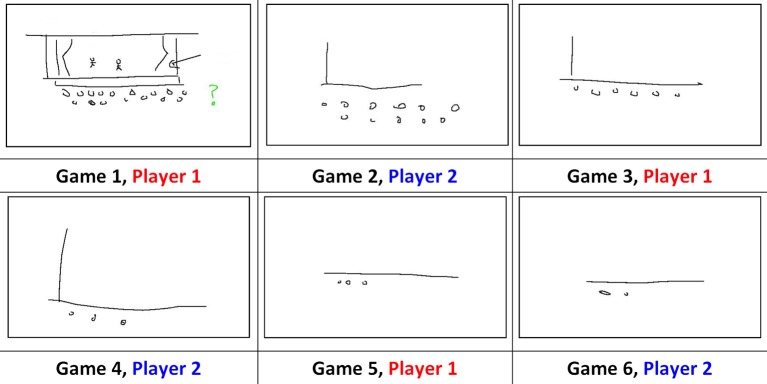
**Sign refinement and alignment for the item Theatre across six games between a pair playing the Pictionary-like task (Figure 1 from Fay and Ellison, 2013)**. Participants alternate directing and matching roles from game to game.

A key finding of relevance to the present study is that participants initially use iconic signs to ground shared meanings, and over subsequent interactions these signs become increasingly aligned, symbolic and language-like (Garrod et al., 2007; Fay et al., 2010; Garrod et al., 2010). In Garrod et al. (2007) participants communicated a set of recurring items to a partner by drawing on a shared whiteboard (e.g., *Art Gallery*, *Drama*, *Theatre*). Much like the game Pictionary™, participants were not allowed to speak or use numbers or letters in their drawings. This procedure forced them to create a new communication system from scratch. As participants repeatedly played the game, the form of their signs changed: for example, at game 1 the sign used to communicate Theater was a visually complex iconic drawing of a theater, including a stage, curtains, actors and an audience, whereas by game 6 it had evolved into a simple symbolic drawing, communicated by a line and two circles. Notice also that the signs produced by each member of the pair became increasingly similar, or aligned over games (see Figure 1). Like spoken referential communication studies, sign refinement is only seen when participants interact with a partner. Repeated drawing without interaction does not lead to such abstraction (in fact, the drawings become more complex; Garrod et al., 2007, 2010).

Experimental-semiotic studies indicate that, when people are prohibited from using their existing language, they use iconic signs to ground shared meanings. Once grounded, the signs become increasingly simplified and aligned, much like spoken language referential communication studies. This process makes the signs easier to execute and comprehend. Given that gesture lends itself more naturally to the production of iconic signs than vocalization, Fay et al. (2013) reasoned that gesture has the potential to provide a superior modality for bootstrapping a communication system from scratch. They tested this prediction in a referential communication study where pairs of participants communicated sets of items (Emotions, Actions, Objects) using non-linguistic vocalization, gesture, or a combination of non-linguistic vocalization and gesture. As predicted, gesture proved more effective (more communication success) and more efficient (less time taken) than vocalization at communicating the different items. Combining gesture with vocalization did not improve performance beyond gesture alone. This finding suggests an important role for gesture in the origin of the earliest human communication systems.

## Present study

Communication is not possible unless people share a common inventory of sign-meaning mappings. The present study tests the extent to which communication modality drives the creation of such an inventory. As in Fay et al. (2013), pairs of participants were assigned to a communication modality (non-linguistic vocalization, gesture, non-linguistic vocalization and gesture combined) and tried to communicate a set of recurring items (Emotions, Actions, Objects) to their partner. Sign alignment was not possible in the Fay et al. (2013) study because participants were allocated to fixed roles (director or matcher) for the duration of the experiment. In the present study participants alternate roles from game to game, allowing them to copy (or not) features of their partners' signs. This simple change in design lets us determine the extent to which partners align their signs.

Our first hypothesis is that communication success will be higher for gesture than for non-linguistic vocalization. Such a result would confirm the findings reported by Fay et al. (2013). Our second hypothesis speaks to the affordance offered by combining modalities. If combining modalities is advantageous because the two modalities offer independent sources of information, we would expect communication success to be higher in the combined modality compared to gesture-alone. While no difference in communication success between gesture and the combined modality was reported by Fay et al. (2013) this may be due to a lack of statistical power. The present study uses almost twice as many participants and double the number of communication games.

The main focus of this paper is alignment. Intuitively, people must establish a mutually shared sign-to-meaning mapping before they can align their sign systems. The extent to which sign-to-meaning mappings are shared is indexed by communication success. Following our first hypothesis (greater communication success in the gestural modality), we therefore expect greater agreement in sign-to-meaning mappings in the gestural modality. Agreement in interpretation, while not enforcing alignment, i.e., use of the same meaning-to-sign mapping, is a prerequisite for the latter. Thus, our third hypothesis is that there will be greater alignment in the gestural modality than in the vocalization modality. Based on our prediction that communication success will be highest in the combined modality, our fourth hypothesis is that alignment will be strongest when both modalities are used.

Our final hypothesis concerns the relationship between communication success and alignment. As discussed above, communication success can be seen as an index of sign-to-meaning agreement, which enables alignment. Evidence of this is seen in a study that established a link between linguistic alignment and performance on a joint cooperative task (Fusaroli et al., 2012). Hypothesis five is that there will be a positive correlation between communication success and sign alignment in each modality.

## Methods

This study received approval from the University of Western Australia Ethics Committee. All participants viewed an information sheet before giving written consent to take part in the study. The information sheet and consent form were both approved by the aforementioned Ethics Committee.

### Participants

Ninety-two undergraduate psychology students (57 females) participated in exchange for partial course credit or payment. Participants were tested in unacquainted pairs, in testing sessions lasting 1 h. All were free of any visual, speech or hearing impairment.

### Task and procedure

Participants completed the task in pairs. Participants were randomly assigned to the role of director or matcher and switched roles at the end of each game, e.g., Participant 1 was the director on Game 1 and Participant 2 was the matcher; on Game 2 Participant 2 was the director and Participant 1 was the matcher, and so on across Games 1–12. Each game consisted of 18 trials. On any trial, the director's task was to communicate a specific item from an ordered list of 24 items (18 target items and 6 distractor items presented on a sheet of A4 paper) that were known to both participants. Items were drawn from three categories (Emotion, Action, Object) and included easily confusable items such as *Tired* and *Sleeping* (see Table 1 for a complete listing of the experimental items). The director's task was to communicate the first 18 items from their list in the given order. On the director's list the first 18 items were always the target items (presented in a different random order on each game). The 18 target items were the same on each game and for each pair of participants. On the director's list the final 6 items were always the distracter items (presented in a different random order on each game). The 6-distractor items were the same on each game and for each pair of participants. Distractor items were included to ensure that matchers could not use a process of elimination to identify the target items. The distracter items were never communicated. The matcher's list was presented in a different random order on a sheet of A4 paper (with all 24 items presented in a different random order). The matcher's task was to indicate the order in which each item was communicated by inserting the trial number beside the relevant item. Participants played the game 12 times with the same partner, using the same item set on each game (i.e., each participant directed 6 times and matched 6 times).

**Table 1:** **The experimental items directors tried to communicate to matchers (distracter items are given in italic)**.

**Emotion**	**Action**	**Object**
Tired	Fleeing	Rock
Pain	Sleeping	Fruit
Angry	Fighting	Predator
Hungry	Throwing	Water
Disgust	Chasing	Tree
Danger	Washing	Hole
*Happy*	*Eating*	*Mud*
*Ill*	*Hitting*	*Rain*

*Target and distracter items were fixed across conditions and throughout the experiment*.

Each pair was randomly allocated to one of three communication modalities: Vocal (*N* = 28), Gesture (*N* = 28) or Combined (gesture plus vocalization) (*N* = 26). In each modality, participants were seated at opposite sides of a round Table 1 meter in diameter. Those in the Vocal modality were told they could make any sounds, and as many sounds (including vocal repetitions) as they wished, but were not permitted to use words. In this modality, participants sat back-to-back, ruling out the use of visual signals. Once the director had communicated each of the 18 target items, the pair swapped roles and the next game began. The new director then communicated the same 18 target items, but in a different random order. This process was repeated until 12 games had been played. Those in the Gesture modality faced one another across the table. All communication was limited to gesture (hand, body and face) and vocalizing was prohibited. Participants were permitted to make any gestures, and as many gestures (including gesture repetitions) as they wished. Participants in the Combined modality followed the same procedure as those in the Gesture modality, but were permitted to vocalize in addition to gesturing. In each modality, matchers indicated to directors they had made their selection by saying “ok,” and then privately inserting the trial number (1–18) next to the selected item. Matchers were only permitted to select an item once.

Irrespective of role, both participants could interact within a trial (e.g., a matcher might seek clarification by frowning or by grunting). As in most human communication studies, participants were not given explicit feedback with regard to their communication success (e.g., Clark and Wilkes-Gibbs, 1986; Garrod and Anderson, 1987; Anderson et al., 1991; Garrod et al., 2007). All communication was recorded using a pair of digital video cameras (one trained on each participant).

## Results

We took two measures of the developing communication systems: effectiveness and alignment. Effectiveness was operationalized as the percentage of items successfully identified by the matcher. Alignment measured the degree to which participants used the same signs as their partner for the same items.

### Effectiveness

**Figure 2:**
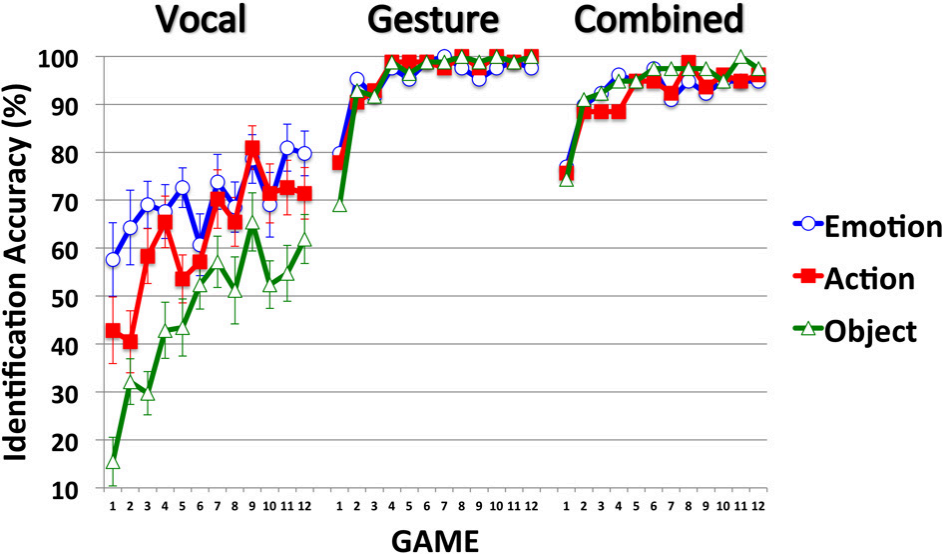
**Mean identification accuracy across Items (Emotion, Action, Object) and Games (1–12), expressed as percentage scores, for participants in the Vocal, Gesture, and Combined modalities**. Error bars indicate the standard errors of the means (included only on items in the Vocal modality to reduce unnecessary clutter).

Effectiveness measures how successful the signs were at identifying their referent. As Figure 2 shows, participants' identification success improved across games 1–12 in all modalities and for each item type (Emotion, Action and Object). In the Gesture and Combined modalities, the different item types were communicated with similar success. In the Vocal modality, Emotion items were more successfully communicated than Action items (in the early games but not in the late games) and Action items were more successfully communicated than Object items (across all Games). Communication effectiveness was very high (and close to ceiling) in the Gesture and Combined modalities, and much lower in the Vocal modality.

For simplicity, and to reduce between-game variance, the factor Games was collapsed into three bins corresponding to Early (1–4), Middle (5–8), and Late (9–12) Games. Participants' mean percent accuracy scores were entered into a mixed design ANOVA that treated Modality (Vocal, Gesture, Combined) as a between-participant factor and Item (Emotion, Action, Object) and Game (Early, Middle, Late) as within. All main effects were significant, as were each of the two-way interactions and the three-way Modality-by-Item-by-Game interaction (see Table 2A).

**Table 2:** **(A) Results of the 3 × 3 × 3 ANOVA that treated Modality (Vocal, Gesture, Combined) as a between-participant factor and Item (Emotion, Action, Object) and Game (Early, Middle, Late) as within-participant factors. Results of the 3 × 3 ANOVAs for each level of Modality: (B) Vocal, (C) Gesture, and (D) Combined**.

	***df***	***F***	***p***	**Partial eta squared**
**(A) OVERALL **3** × **3** × 3 ANOVA**				
Modality	2, 38	123.02	<0.001	0.87
Item	2, 76	22.17	<0.001	0.37
Game	2, 76	70.64	<0.001	0.65
Modality × item	4, 76	25.93	<0.001	0.58
Modality × game	4, 76	8.46	<0.001	0.31
Item × game	4, 152	6.52	<0.001	0.15
Modality × item × game	8, 152	2.42	=0.017	0.11
**(B) VOCAL**				
Item	2, 26	39.53	<0.001	0.75
Game	2, 26	28.55	<0.001	0.69
Item × game	4, 52	5.27	<0.001	0.29
**(C) GESTURE**				
Item	2, 26	0.11	=0.90	0.01
Game	2, 26	48.98	<0.001	0.79
Item × game	4, 52	1.60	=0.19	0.11
**(D) COMBINED**				
Item	2, 24	0.45	=0.64	0.04
Game	2, 24	25.40	<0.001	0.68
Item × game	4, 48	0.96	=0.44	0.07

To understand the 3-way interaction we ran three separate Item-by-Game ANOVAs for each level of Modality (Vocal, Gesture, Combined). The 3-way interaction can be explained by the Item-by-Game interaction in the Vocal modality, and the sole main effect of Game in the Gesture and Combined modalities (Tables 2B–D, respectively). Although communication success improved across games for each item type in each modality, in the Vocal modality the different items were communicated with different levels of success. In the Early games, Emotion items were more successfully communicated than Action items, and Action items were more successfully communicated than Object items. By the late games, Emotion and Action items were communicated with equal success, and both were communicated with greater success than Object items. In contrast, the different item types were communicated with similar success in both the Gesture and Combined modalities.

In support of Hypothesis 1, and as observed by Fay et al. (2013), communication success was higher for each item type in the Gesture and Combined modalities than in the Vocal modality: Emotion [*F*s_(1, 26/25)_ > 28.12, *p*s < 0.001, η^2^_*p*_s > 0.53], Action [*F*s_(1, 26/25)_ > 65.54, *p*s < 0.001, η^2^_*p*_s> 0.72] and Object items [*F*s_(1, 26/25)_ > 226.23, *p*s < 0.001, η^2^_*p*_s > 0.90]. Hypothesis 2, which predicted higher communication success in the Combined modality, was not supported. Communication success was comparable across the Gesture and Combined modalities for Emotion and Object items [*F*s_(1, 25)_ < 1.09, *p*s < 0.31, η^2^_*p*_s < 0.04]. However, Gesture proved more successful than the Combined modality at communicating Action items [*F*s_(1, 25)_ = 4.84, *p*s = 0.037, η^2^_*p*_s = 0.16]. Thus, with more statistical power, the null effect reported by Fay et al. (2013) reached statistical significance in the present study.

Gesture is a more effective means of communication than vocalization, and combining gesture with vocalization does not improve communication success beyond gesture alone. In fact, it may make it worse.

### Alignment

**Figure 3:**
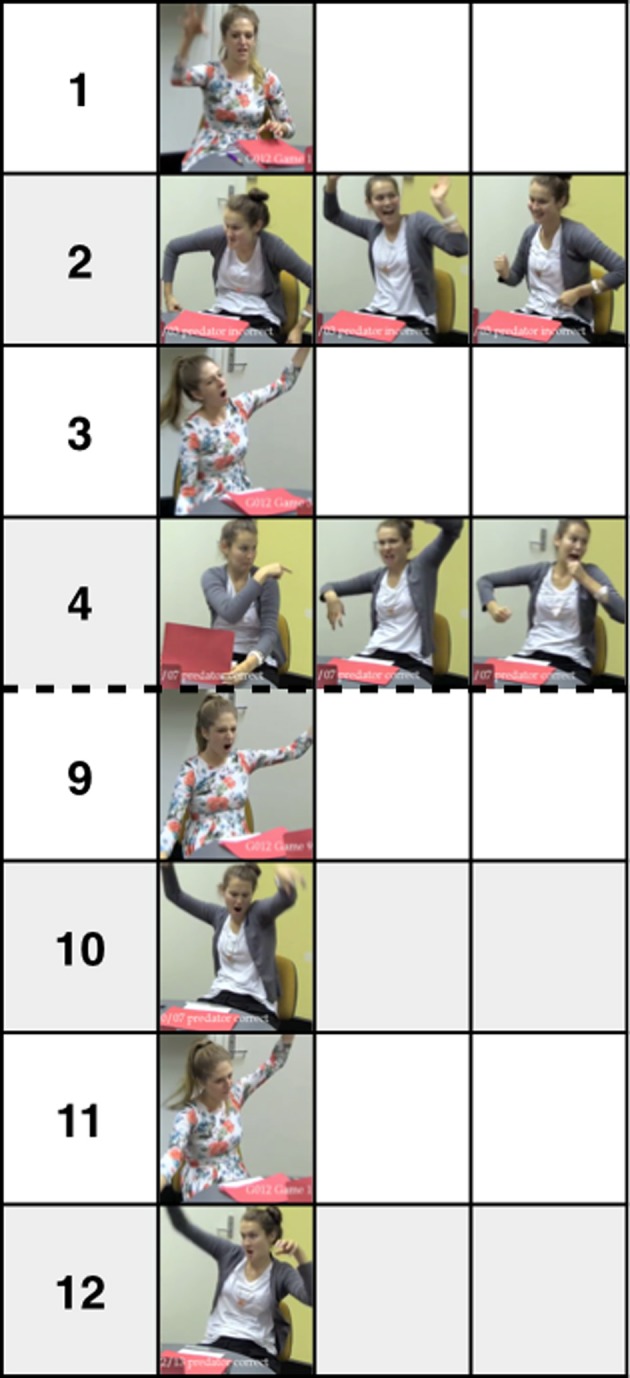
**Signs used by a pair in the Gesture modality to communicate the object “predator” at Games 1–4 (Early) and 9–12 (Late)**. Game number is given in the leftmost column. At Game 1 Director A claws at the air (correctly identified by partner). At Game 2 Director B mimes a hulking movement, with her arms out to the side. Next she throws her arms up in fright before miming a running action (incorrectly identified). At Game 3 Director A copies Director B; she throws her arms in the air and mimes walking like a hulk (incorrectly identified). At Game 4 Director B points over her shoulder, mimes walking like a hulk, then mimes running (correctly identified). Communication is simple, aligned and successful from Game 9: both partners communicate “predator” by raising their arms in their air to mime a hulk walking.

An illustrative example of communication from a pair of participants in the Gesture modality, sampled from the early (1–4) and late games (9–12) is given in Figure 3. Initially a variety of different signs were used to communicate the object “predator.” Eventually the partners aligned on the same simplified sign.

**Figure 4:**
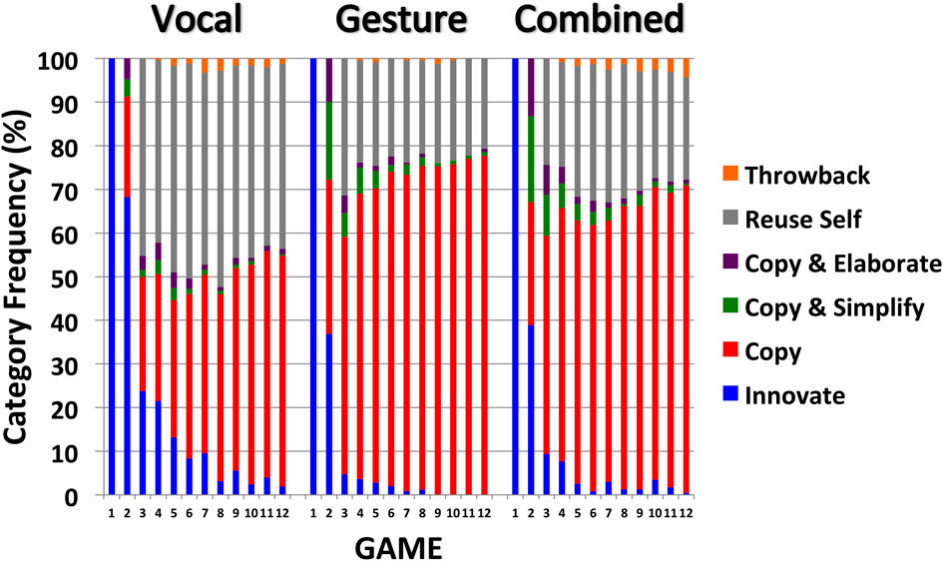
**Mean frequency, expressed as percentage scores, of Innovate, Copy, Copy, and Simplify, Copy and Elaborate, Reuse Self and Throwback signs across Games 2–12 for participants in the Vocal, Gesture and Combined modalities**. Error bars indicate the standard errors of the means.

A bespoke coding scheme was developed to elucidate the process through which pairs of participants establish a shared communication system. The coding scheme was designed to assess sign variation and the extent to which pairs of participants were able to negotiate a stable and shared sign for each meaning over the course of the experiment. Broadly, we predict that sign stability/sharedness will increase across games in each modality. The coding scheme was applied to the signs produced by directors in each modality, as they communicated the 18 different target items across games 1–12. Each sign was coded into one of the following categories: Innovate (new, previously unseen sign for this item), Copy (replication of partner's sign for the same item from the immediately prior game), Copy and Simplify (simplified version of partner's sign for the same item from the immediately prior game), Copy and Elaborate (more complex version of partner's sign for the same item from the immediately prior game), Reuse Self (participant reuses a sign for the same item from their prior turn as director), and Throwback (participant uses a sign for the same item from an earlier game, but not one from their partner's immediately prior turn as director, or from their own immediately prior turn as director). The changing frequencies of the different sign categories are shown in Figure 4 (collapsed across the different item types). Video examples from each modality are available at http://comlab.me/ComLab/GestureBeatsVocal.html.

Innovation is the only option at Game 1 as there are no earlier signs to copy. Hence, there is 100% sign Innovation at Game 1 in each modality. From this point onwards, sign Innovation decreases dramatically across games. This decrease in Innovation is most strongly observed in the Gesture and Combined modalities, compared to the Vocal modality. As Innovation decreases, sign Copying increases over games. Sign Copying is more strongly observed in the Gesture and Combined modalities (78 and 71% respectively by Game 12) compared to the Vocal modality (52%). Sign Copy and Simplify was prominent at Game 2 in the Gesture and Combined modalities (18 and 20%, respectively) and was almost absent by Game 12 (<1%). Copy and Elaborate was less frequent but showed a similar pattern (10 and 13%, respectively, at Game 2 and <1% by Game 12). Sign Copy was less frequent in the Vocal modality (52% at Game 12), as was Copy and Simplify (4% at Game 2) and Copy and Elaborate (5% at Game 2). Participants in the Vocal modality frequently Reused the sign they produced on their prior turn as director (42% at Game 12, compared to 21 and 23% in the Gesture and Combined modalities). Throwbacks were too infrequent to compare (occurring on only 1.2% of trials across Games 2–12). The more frequent sign Copying observed in the Gesture and Combined modalities indicates that the signs were more shared, or aligned, in these modalities, compared to the Vocal modality.

**Figure 5:**
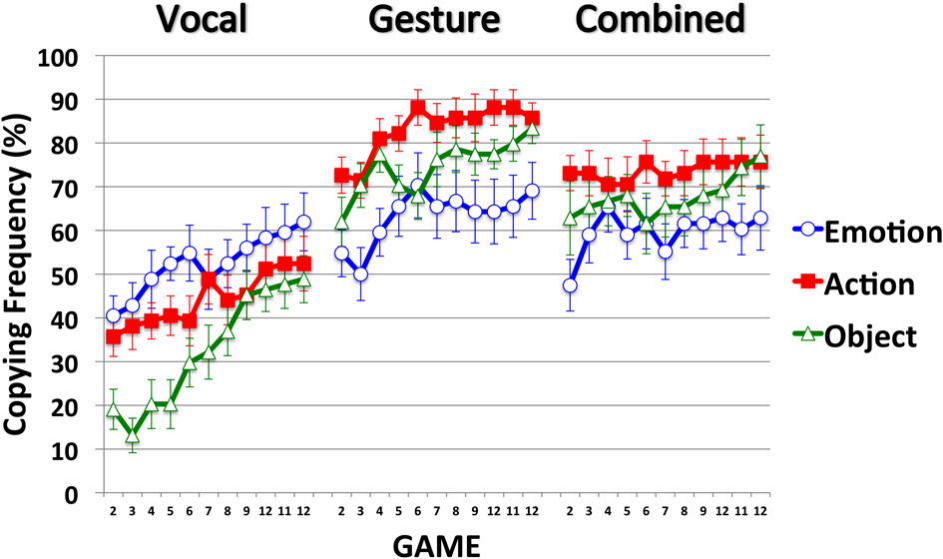
**Mean copying frequency, expressed as percentage scores, of signs across Items (Emotion, Action, Object) and Games (2–12) for participants in the Vocal, Gesture and Combined modalities**. Error bars indicate the standard errors of the means.

We tested this observation by comparing the overall frequency of Sign Copying (by combining the Copy, Copy and Simplify and Copy and Elaborate categories) across the different modalities. Game 1 was not included in the analysis as sign Copying was not possible. As Figure 5 shows, sign copying increased across games in each modality, and for each item type. Sign copying is comparable across modalities for Emotion items, but is higher in the Gesture and Combined modalities for Action and Object items.

The factor Game was again collapsed into three bins corresponding to Early (2–4), Middle (5–8), and Late (9–12) Games. Participants' mean percent Copying scores were entered into a mixed design ANOVA that treated Modality (Vocal, Gesture, Combined) as a between-participants factor and Item (Emotion, Action, Object) and Game (Early, Middle, Late) as within. This returned main effects for Modality, Item and Game [*F*s_(2, 38/76)_ < 6.41, *p*s < 0.003, η^2^_*p*_s> 0.14]. There was also a Modality-by-Item and Modality-by-Game interaction [*F*s_(2, 76)_ < 4.90, *p*s < 0.001, η^2^_*p*_s > 0.21]. No other effects reached statistical significance [*F*s < 2.08, *p*s > 0.09, η^2^_*p*_s < 0.05].

As Figure 5 shows, sign alignment in the Vocal modality mirrors identification accuracy: stronger alignment on Emotion items followed by Action and Object items. A different pattern is observed in the Gesture and Combined modalities where stronger alignment is seen for Action items followed by Objects and Emotion items. More importantly, pairwise comparisons indicate a similar level of alignment for Emotion items across the different modalities [*t*s_(26/25)_ < 1.44, *p*s > 0.16, *d*s < 0.542], but stronger alignment for Action and Object items in the Gesture and Combined modalities compared to the Vocal modality [*t*s_(26/25)_ > 4.55, *p*s < 0.001, *d*s > 1.75]. A similar level of alignment was observed for each item type in the Gesture and Combined modalities [*t*s_(25)_ < 1.69, *p*s > 0.10, *d*s < 0.65]. Thus, the Modality-by-Item interaction can be explained by a similar level of alignment across modalities for Emotion items, and stronger alignment for Action and Object items in the Gesture and Combined modalities (compared to the Vocal modality).

The Modality-by-Game interaction is explained by the strong increase in sign copying across games in the Vocal modality [*F*_(2, 26)_ = 22.82, *p* < 0.001, η^2^_*p*_ = 0.64] and Gesture modality [*F*_(2, 26)_ = 13.17, *p* < 0.001, η^2^_*p*_ = 0.50] and the weaker, marginal increase in sign copying in the Combined modality [*F*_(2, 24)_ = 2.95, *p* = 0.057, η^2^_*p*_ = 0.21]. Pairwise comparisons indicate that sign alignment is stronger for Early, Middle and Late games in the Gesture and Combined modalities, compared to the Vocal modality [*t*s_(26/25)_ > 2.69, *p*s < 0.013, *d*s > 1.04]. Sign alignment scores were similar in the Gesture and Combined modalities [*t*s_(25)_ < 1.74, *p*s > 0.094, *d*s < 0.67].

In summary, there was greater sign alignment when participants could use gesture to communicate. This finding supports Hypothesis 3. Hypothesis 4, that sign alignment will be stronger in the Combined modality, was not supported. In fact, sign alignment increased more strongly in the Gesture modality compared to the Combined modality.

### Effectiveness and alignment

**Figure 6:**
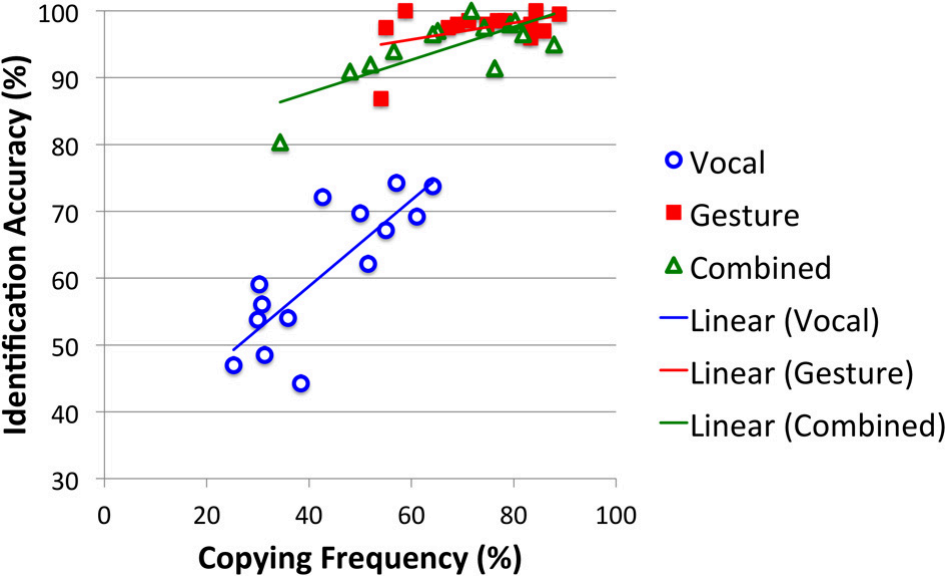
**Correlation between Identification Accuracy (mean percent of items correctly identified across games 2–12) and Sign Copying (mean percent of signs copied across games 2–12) for participants in the Vocal, Gesture and Combined modalities**.

To what extent are communication effectiveness and sign alignment linked? Hypothesis 5 predicts a positive correlation between the two. This would be consistent with communication success promoting sign alignment and/or sign alignment promoting communication success. To determine if a relationship exists, participants' mean identification accuracy scores (collapsed across games 2–12) were correlated with their mean copying scores (collapsed across games 2–12). A strong positive correlation was observed in the Vocal [*r*_(14)_ = 0.81, *p*_one−tailed_ < 0.001] and Combined modalities [*r*_(13)_ = 0.75, *p*_one−tailed_ = 0.001], and a moderate correlation was observed in the Gesture modality [*r*_(14)_ = 0.45, *p*_one−tailed_ = 0.055]. The correlations in the Gesture and Combined modalities are all the more remarkable given the lack of variation in identification accuracy scores (due to the near ceiling effect; see Figure 6). This pattern supports Hypothesis 5.

## Discussion

The present study experimentally tested the influence of modality (vocal, gesture, or a combination of the two) on how people establish a shared communication system from scratch when they cannot use an existing language system. Gesture proved to be a more effective means of communication than non-linguistic vocalization, supporting Hypothesis 1^1^. Hypothesis 2, that combining the two modalities would prove more effective than gesture alone, was not supported. In fact, Gesture was comparable to the Combined modality for Emotion and Object items, and was more successful at communicating Action items.

The primary motivation behind the present study was to test how modality affects the establishment of a shared inventory of signs. This shared inventory arises via progressive sign alignment (Pickering and Garrod, 2004). Gesture enabled stronger sign alignment than Vocalization for Action and Object items, but not for Emotion items, partly supporting Hypothesis 3. Hypothesis 4, that combining the two modalities would produce stronger alignment than gesture alone, was not supported. In fact, the increase in sign alignment across games was stronger for Gesture alone than for the Combined modality. Hypothesis 5 predicted a positive correlation between communication success and sign alignment. Consistent with a link between linguistic alignment and task performance (Fusaroli et al., 2012), a positive correlation between communication success and sign alignment was returned for each modality. Of course, causality cannot be determined: communication success may promote sign alignment or sign alignment may promote communication success, or both. We suspect causality acts in both directions.

### Why are communication success and sign alignment higher for gesture than for vocalization?

Among modern day humans, with modern brains and mastery of at least one spoken language, the present study demonstrates the superiority of gesture over non-linguistic vocalization as a solution to the Babel problem. In this context gesture is a more precise modality of communication than non-linguistic vocalization. We believe this precision arises from its greater affordance of motivated signs: iconic signs that communicate through resemblance, or indexical signs that communicative via a natural association between sign and referent. For Vocalization, the link between sign and referent tends to be arbitrary, that is, symbolic, with the exception of a small inventory of onomatopoeic and sound-symbolic expressions (see Shintel and Nusbaum, 2007). For example, participants in the Gesture modality could close their eyes and pretend sleep to communicate *Tired* (a natural index of tiredness), clench their fist and pantomime throwing a punch to communicate *Fighting* (an iconic representation) or peel an imaginary banana to communicate *Fruit* (an indexical representation). These motivated relationships between sign and referent are much less obvious for Vocalization. They do exist for some Emotion items, for example, making yawn noises to communicate *Tired* (a vocal index of tiredness), but are mostly absent for Action and Object items. For instance, it's hard to imagine a motivated vocalization that could be used to communicate *Chasing* or *Mud*. Our data support this: in the Combined modality, vocalization was added to gesture on 54% of trials for Emotion items, 26% of trials for Object items and 14% of trials for Action items (and remained stable across games).

Our study suggests that affordances of motivated signs are essential to bootstrapping a set of shared sign-meaning mappings when people cannot draw on a pre-existing inventory of shared conventional signs. Once the sign-meaning mappings have been grounded, interlocutors can reduce the complexity of the signs—causing them to evolve into more symbol-like forms (Garrod et al., 2007, 2010)—and align their signs. Both processes reduce the cost of sign production and comprehension (Pickering and Garrod, 2004, 2013). These local interactive processes underpin the propagation of a shared inventory of conventional signs in larger populations, as shown in computer simulations (Steels, 2003; Barr, 2004; Tamariz et al., under review), natural spoken language studies (Garrod and Doherty, 1994), experimental semiotic studies (Fay et al., 2008, 2010; Fay and Ellison, 2013) and naturalistic studies of recently formed sign languages (Goldin-Meadow et al., under review; Kegl et al., 1999).

Returning to theories of the origin of language, our results suggest a strong role for gesture due to its affordance of motivated signs. In the absence of a conventional language, it is unlikely that our ancestors would have passed up the opportunity to use motivated signs, in particular gesture, to get their point across. This is to not to rule out a multimodal, “catch-as-catch-can” account (Bickerton, 2007, p. 512), far from it: when permitted, participants often used vocalization in combination with gesture, especially for Emotion items (54% of trials in the Combined modality). The productive use of vocalization as an index of emotions (see also Sauter et al., 2010) fits with our position that motivated signs are likely to have played an important role in establishing the earliest human communication systems. However, it is important to be clear that in the present study vocalization played a supporting role, always occurring in the company of gesture and not replacing gesture. Gesture, we propose, played the primary role in bootstrapping the earliest human communication systems on account of its affordance of motivated signs. Today, the vocal modality is primary and gesture plays a supporting role. The dynamics of the rise of predominantly vocal language, and the reasons for it, are targets for future research (see Goldin-Meadow and McNeill, 1999; Corballis, 2002; Corballis, for some suggestions such as the affordance of vocalization for communication in the dark).

### Why is gesture better than gesture plus vocalization at communicating action items?

The finding that Gesture alone was more successful at communicating Action items than the Combined modality warrants further consideration. One candidate explanation is that participants were distracted by the auditory information conveyed in the Vocal modality (Spence et al., 2000). This explanation is plausible because Vocal-only communication is less precise than Gesture-only communication in the present study. If information conveyed in the vocal channel acts as a distractor from information conveyed in the visual channel, we would expect a negative correlation between vocalization frequency and communication success. That is, more frequent vocalization will be associated with lower communication success. Participants' mean vocalization frequency (percent of trials in which vocalization occurred in addition to gesture collapsed across games 1–12) was correlated with their mean communication success. A moderate negative correlation was returned [*r*_(13)_ = −0.39, *B* = −0.138, *p*_one−tailed_ = 0.095], indicating that more frequent vocalization is associated with lower communication success for Action items. Although a similar negative correlation was observed for Object items [*r*_(13)_ = −0.48, *B* = −0.075, *p*_one−tailed_ = 0.045], its gradient is shallower compared to that of Action items, meaning that the negative impact of vocalization on communication success was less strongly felt. The correlation for Emotion items did not approach statistical significance [*r*_(13)_ = −0.13, *B* = −0.030, *p*_one−tailed_ = 0.339].

Why did vocalization negatively impact communication success for Action items? More than Object or Emotion items, Action items offer an opportunity for embodiment in the Gesture modality (Lakoff and Johnson, 1999; Hostetter and Alibali, 2008). By taking a character viewpoint, participants can simulate the action as the sign: to communicate *Throwing* the participant can extend their right arm back and mime the throwing of a ball. Embodied action is less direct for Emotion items, which are internal states, and Object items, which have no direct human role to take (although some participants pantomimed a human interaction with the object). The infrequent addition of vocalization when communicating Action items in the Combined modality (14% of trials) reflects the intrinsic fit between gesture and actions. This fit is reinforced by Action items exhibiting the strongest levels of sign alignment in the Gesture modality, compared to the other item types (see Figure 5). Against this natural fit between gesture and actions, supplementary vocalizations distract the matcher from a channel that is ideally suited to the communication of actions.

### Experimental gesture creation compared to naturalistic gesture creation

Our study has some limitations, the most important of which is that our participants have modern day brains and already speak a language. The second is that our participants are creating labels out of context, which is not likely to be the way language emerges on the ground. Finally, we ask our participants to create words, but we do not ask them to string those words together, that is, to create sentences. Studies of naturalistic language creation in homesigners address some, but not all, of these limitations. As mentioned earlier, homesigners are individuals whose profound hearing losses prevent them from acquiring the spoken language that surrounds them, even when given intensive instruction in speech. They are, in addition, born to hearing parents who do not expose them to a conventional sign language. Under these circumstances, we might expect that a homesigner would not communicate at all. But homesigners do communicate, and they use gesture to do so (Goldin-Meadow, 2003).

Homesigners thus do not have usable input from a conventional language model and are truly creating language from scratch (although they do have modern day brains). Moreover, the gestures homesigners create are all used in a naturalistic context. Like the participants in our study, young homesigners use iconic gestures to refer to actions. However, they prefer to use pointing gestures, rather than iconic gestures, to refer to objects (they rarely refer to emotions, but neither do young children learning conventional language). Over time, homesigners use iconic gestures more and more often to refer to objects as well as actions, and they develop morphological devices to distinguish between the two uses (Goldin-Meadow et al., 1994). Not surprisingly, because they are communicating with hearing individuals who do not share their gesture systems, homesigners rarely produce gestures whose forms are not transparently related to their referents; that is, they rarely produce non-iconic gestures. For the same reason, their gestures do not lose their iconicity over time. Nevertheless, these iconic gestures are combined with other gestures to form structured sentences. Homesigners combine their pointing gestures (and later their iconic gestures referring to objects) with iconic gestures referring to actions, and use these gesture sentences to communicate about the here-and-now and the non-present, to make generic statements, to tell stories, to talk to themselves, and even to refer to their own gestures—that is, to serve the central functions of language (Goldin-Meadow, 2003). The fact that homesigners begin the process of language creation by using gesture to convey actions fits nicely with our finding that gesture affords an easily accessible way to convey action, and suggests that our experimental paradigm is capturing an early stage of an important aspect of language creation.

In addition to creating gestures in a naturalistic context, homesigners also differ from our participants in that they are interacting with hearing individuals who have no interest in creating a shared gesture system with them. Homesigners in the U.S. are typically born to hearing parents who would like their deaf children to learn to speak; they therefore often do not learn sign language themselves and rarely gesture to their children without talking at the same time (Flaherty and Goldin-Meadow, 2010). The gestures they produce are thus co-speech gestures, which are qualitatively different in form from homesign (Goldin-Meadow et al., 1996). In other words, the homesigners' parents do *not* align their gestures with their children's gestures (Goldin-Meadow and Mylander, 1983). Interestingly, although homesigners display many of the grammatical features of natural language in their gestures, their gestures do not form a stable lexicon in the same way that our participants' gestures do. Goldin-Meadow et al. (under review) studied adult homesigners in Nicaragua and found that they used different gestures from each other to label the same object, which is not surprising given that the homesigners did not know one another. More importantly from our point of view, each individual homesigner used a variety of gestures to label a single object and was not consistent within him or herself. The homesign data thus support the conclusions from our study—that alignment between speakers is essential for a lexicon to stabilize.

## Conclusion

The Tower of Babel story asks if people can communicate when they do not share a common language. The present study experimentally tests the affordances offered by vocalization and gesture when creating a common inventory of signs from scratch. Gesture outperformed non-linguistic vocalization both in terms of communication success and in terms of the creation of a common inventory of sign-meaning mappings. Combining vocalization with gesture did not improve performance beyond gesture alone; in fact, it sometimes proved deleterious. We argue that the benefit of gesture lies in its ability to communicate through motivated signs, and this makes it an excellent modality for language creation.

### Conflict of interest statement

The authors declare that the research was conducted in the absence of any commercial or financial relationships that could be construed as a potential conflict of interest.

## Footnotes

^1^Gesture might be more effective (communication success) than vocalization because vocalization suffers greater interference from participants' first (spoken) language. This is possible, although it is equally possible that communication success in the vocalization-only condition was facilitated by participants' first (spoken) language. An issue for an interference explanation is that the different item types (emotion, action, object) showed a differential pattern of communication success. General interference from an already established vocal language would predict a similar performance decrement in the vocalization-only modality for the different item types relative to the gesture modality. Further research with deaf signers or bimodal bilinguals (e.g., English-ASL) is needed to make a definite determination about whether performance on the task is affected by participants' existing language system.
